# Notch signalling patterns retinal composition by regulating *atoh7* during post-embryonic growth

**DOI:** 10.1242/dev.169698

**Published:** 2018-11-09

**Authors:** Alicia Pérez Saturnino, Katharina Lust, Joachim Wittbrodt

**Affiliations:** 1 Centre for Organismal Studies, Heidelberg University, Heidelberg 69120, Germany; 2 Heidelberg Biosciences International Graduate School (HBIGS), Heidelberg 69120, Germany

**Keywords:** Notch, Atoh7, Retinal progenitors, Post-embryonic growth, Cell specification, Medaka

## Abstract

Patterning of a continuously growing naive field in the context of a life-long growing organ such as the teleost eye is of high functional relevance. Intrinsic and extrinsic signals have been proposed to regulate lineage specification in progenitors that exit the stem cell niche in the ciliary marginal zone (CMZ). The proper cell-type composition arising from those progenitors is a prerequisite for retinal function. Our findings in the teleost medaka (*Oryzias latipes*) uncover that the Notch-Atoh7 axis continuously patterns the CMZ. The complement of cell types originating from the two juxtaposed progenitors marked by Notch or Atoh7 activity contains all constituents of a retinal column. Modulation of Notch signalling specifically in Atoh7-expressing cells demonstrates the crucial role of this axis in generating the correct cell-type proportions. After transiently blocking Notch signalling, retinal patterning and differentiation is re-initiated *de novo*. Taken together, our data show that Notch activity in the CMZ continuously structures the growing retina by juxtaposing Notch and Atoh7 progenitors that give rise to distinct complementary lineages, revealing coupling of *de novo* patterning and cell-type specification in the respective lineages.

## Abstract (precis)

**Summary:** Mutually exclusive activity of Notch and Atoh7 in the ciliary marginal zone gives rise to two distinct lineages resulting in specification of the full complement of cell types in medaka retina.

## INTRODUCTION

The central nervous system (CNS) presents an extraordinary diversity of neuronal cell types. Even now, the exact number of distinct neuronal cell types is unclear. Moreover, their lineage specification from a common progenitor pool is also very complex (Edlund and Jessell, 1999; Pearson and Doe, 2004). The retina, even though it is part of the CNS, has a relatively simple cellular composition, which has been extensively studied (Bassett and Wallace, 2012). It consists of six neuronal cell types and one glial cell type, which are distributed into three nuclear layers: the outer nuclear layer (ONL) containing the rod and cone photoreceptors (PRCs); the inner nuclear layer (INL) where bipolar cells (BCs), amacrine cells (ACs), horizontal cells (HCs) and Müller glia (MG) cells are located; and the ganglion cell layer (GCL) where the retinal ganglion cells (RGCs) as well as some ACs reside. Its well-characterized structure, together with its accessibility and easy manipulation, make the retina a particularly suitable tissue in which to study the principles of lineage specification.

During lineage specification in retinal development, intrinsic as well as extrinsic factors influence the progression of progenitors through different competence states to achieve the production of the different cell types (Hufnagel and Brown, 2013; Livesey and Cepko, 2001). This process continues throughout life in constantly growing organisms, such as fish and amphibians, and is supported by stem cells, which reside in the most peripheral domain of the retina, the ciliary marginal zone (CMZ) (Johns and Easter, 1977; Hollyfield, 1968). Similarly to embryonic development, this pool of multipotent stem cells gives rise to the whole spectrum of retinal cell types during post-embryonic growth (Centanin et al., 2011, 2014; Wan et al., 2016). However, how lineage specification and patterning are coordinated in a continuously growing organ remains elusive.

Post-embryonic growth shows some marked differences from embryonic development. Whereas new structures and organs need to be formed during embryonic development, already existing functional structures need to be expanded during post-embryonic growth. The Notch signalling pathway has been previously identified as extrinsic factor influencing cell-fate decisions during retinal development (Andreazzoli, 2009; Livesey and Cepko, 2001). Despite extensive studies in retinal development, little is known about cell specification of post-embryonic retinal stem and progenitor cells. The transmembrane receptor Notch as well as other components of the Notch signalling pathway have been reported to be expressed in the CMZ in frogs and fish, suggesting a role in retinal post-embryonic growth (Coffman et al., 1990; Dorsky et al., 1995; Ohnuma et al., 2002; Perron et al., 1998; Raymond et al., 2006). However, the nature of this role has not yet been addressed.

The potential implication of Notch signalling in cell-fate specification during retinal post-embryonic growth is strongly supported by the classical role of Notch signalling in neural development: patterning regulation and cell-fate determination (Louvi and Artavanis-Tsakonas, 2006). Notch signalling is known to regulate tissue diversification by generating a mosaic pattern: Notch signalling propagates in an equipotent tissue, generating a binary pattern, where adjacent cells differ from each other with respect to the activation of the pathway (lateral inhibition). The activation of the pathway occurs when the transmembrane receptor Notch binds its ligand Delta, a protein located in the membrane of the neighbouring cell. This binding triggers proteolytic activity on the receptor releasing its intracellular domain, which translocates into the nucleus and regulates gene expression (Fiúza and Arias, 2007). Notch signalling downstream target genes, Hairy/E(spl)-related factors (Her factors in fish, Hes in mouse) function as transcriptional repressors (Borggrefe and Oswald, 2009). Among their targets, Her factors have been shown to act on basic helix-loop-helix (bHLH) proneural factors (Kageyama et al., 2007). Eventually, this results in a salt-and-pepper pattern of proneural gene expression (Lai, 2004; Neves et al., 2013). Among other bHLH factors, *atoh7* expression has been previously shown to be negatively regulated by Her factors during development (Maurer et al., 2014; Schneider et al., 2001). The role of Atoh7 in retinal development has been previously described in teleost fish. It has been shown to be necessary and sufficient for the development of RGCs (Kanekar et al., 1997; Kay et al., 2001). Atoh7-positive progenitors also give rise to ACs, HCs and PRCs during retinal development (Poggi et al., 2005). Interestingly, *atoh7* has been also shown to be expressed in the progenitor area of the post-embryonic teleost retina (Lust et al., 2016). However, the role for Notch signalling as well as its crosstalk with *atonal* genes in retinal post-embryonic growth is still unknown.

Here, we show that Notch signalling is active in a subset of progenitors in the transit-amplifying zone of the CMZ in the Japanese rice fish medaka (*Oryzias latipes*). This progenitor population is fate-restricted to BCs, ACs and MG cells. Moreover, Notch signalling activation shows a mutually exclusive pattern with the expression of the bHLH transcription factor Atoh7. Manipulation of Notch signalling by targeted activation as well as its chemical inhibition demonstrate its crucial role in generating the correct cell-type proportions within the Atoh7 lineage. All this occurs continuously *de novo* in the CMZ where, after transient Notch inhibition, the Notch-Atoh7 axis is re-initiated from scratch and maintained thereafter. Our data provide mechanistic insight into how a growing organ is patterned continuously *de novo* and how this two-dimensional patterning, the juxtaposition of Notch and Atoh7 cells in the CMZ, impacts on the third dimension of cell-type composition by distinct lineage specification.

## RESULTS

### Notch signalling is active in a subset of retinal progenitors in the post-embryonic retina in medaka

Notch signalling is known to be active in MG cells and the transit-amplifying zone of the CMZ in the zebrafish post-embryonic retina (Link and Darland, 2001; Raymond et al., 2006). Its role in MG cells, which are the retinal stem cells responsible for retinal regeneration in zebrafish, has been extensively studied (Wan and Goldman, 2017; Wan et al., 2012). However, the function of Notch signalling in lineage specification in the transit-amplifying zone of the CMZ is still unknown. We addressed this in the medaka retina. Here, retinal stem cells residing in the CMZ have been recently characterized: they have been shown to be multipotent and the transcriptional network regulating their stemness has also been identified (Centanin et al., 2011, 2014; Reinhardt et al., 2015).

**Fig. 1.**
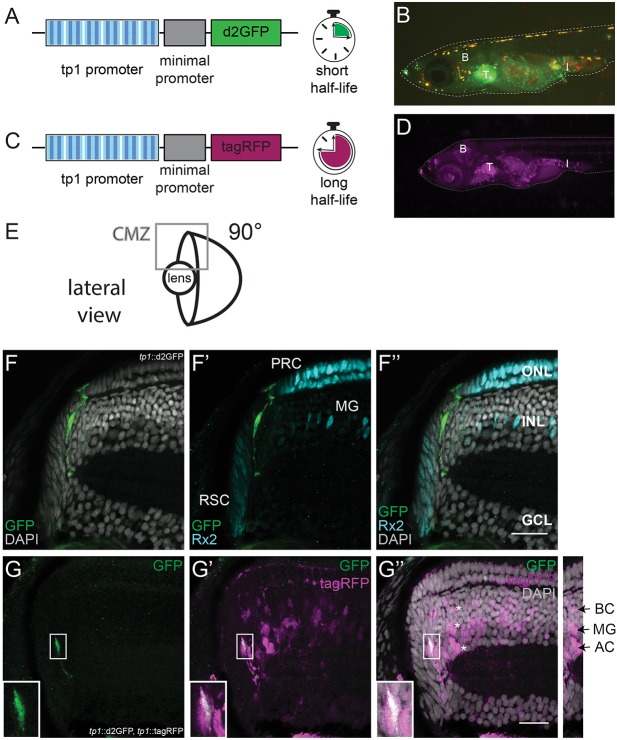
**Notch signalling is active in a subset of retinal progenitors, which give rise to MG cells, ACs and BCs during retinal post-embryonic growth in medaka.** (A) The *tp1-MmHbb::*d2GFP (*tp1::*d2GFP) Notch signalling reporter contains six copies of the *tp1* promoter, a Notch-responsive promoter (blue striped boxes). Each *tp1* promoter contains two RBP-Jk-binding sites (dark blue stripes). The *tp1* promoter is followed by a minimal promoter (mouse β globin) and a destabilized GFP (d2GFP), which has a short half-life. (B) The *tp1::*d2GFP reporter line shows Notch signalling activation in various tissues such as brain (B), thymus (T) and intestine (I) in hatch medaka. (C) The *tp1-MmHbb::*tagRFP (*tp1::*tagRFP) Notch signalling reporter contains the *tp1* Notch-responsive promoter followed by a tagRFP, a very stable red fluorescent protein with a long half-life. (D) The *tp1::*tagRFP reporter line shows Notch signalling activation in hatch medaka in the same tissues as the previously described green reporter line: brain (B), thymus (T) and intestine (I). (E) Schematic of the retina from a lateral view (90°). The CMZ is indicated with a square, which corresponds to the area that is shown in F-G″. (F-F″) Notch signalling (green) is active in a subset of retinal progenitors but it is active in neither RSCs nor differentiated cells (PRCs and MG cells), here labelled with Rx2 (cyan). Nuclear labelling with DAPI is in grey. *n*=10 fish. (G-G″) The two Notch reporter lines (*tp1::*d2GFP in green and *tp1::*tagRFP in magenta) show overlapping activation only in the progenitor area (bottom left insets); 66.14±18.07% (s.d.) of the GFP-positive cells are also tagRFP positive (*n*=8 retinae, 41 Notch reporter-positive cells). Owing to label retention, the *tp1::*tagRFP reporter line is also visible in differentiated cells (asterisks) derived from Notch-positive progenitors: MG cells, ACs and BCs. Scale bar: 20 μm.

To visualize active Notch signalling in the post-embryonic retina in medaka, the previously characterized *tp1-MmHbb::*d2GFP (*tp1::*d2GFP) Notch signalling reporter (Clark et al., 2012; Lust et al., 2016) was used. The reporter construct carries six copies of the *tp1* promoter, a Notch-responsive promoter containing 2 RBP-Jk-binding sites, followed by a minimal promoter (mouse beta globin) and a destabilized GFP (d2GFP) (Fig. 1A). The *tp1::*d2GFP reporter line showed Notch signalling activation in various tissues, such as thymus, brain and intestine, in hatch medaka (8 days post fertilization at 28°C) (Fig. 1B). The Notch signalling activation pattern is highly conserved and activation in these tissues has been previously reported in other organisms from flies to mouse (Bajoghli et al., 2009; Bigas and Espinosa, 2012; Bray, 2016; Siebel and Lendahl, 2017). In addition, we generated a second Notch reporter line for short-term lineage analysis: *tp1-MmHbb::*tagRFP (*tp1::*tagRFP). This line carries the same construct as the *tp1::*d2GFP line but the short-lived d2GFP is replaced by a tagRFP (Fig. 1C). TagRFP is not targeted for degradation and therefore has a long half-life allowing short-term lineage tracing due to label retention (Merzlyak et al., 2007). The *tp1::*tagRFP reporter line showed activation in the same tissues as the *tp1::d2GFP* line, including the brain, the thymus and the intestine in a medaka hatchling (Fig. 1D).

In the retina, we detected Notch signalling activation in a subset of progenitors (Fig. 1E-F″). Retinal progenitors are located in the transit-amplifying zone of the CMZ between the retinal stem cells (RSCs) and the central differentiated retina. RSCs are labelled by antibody staining against the transcription factor retinal homeobox gene two (Rx2) (Reinhardt et al., 2015). They are located in the most peripheral part of the retina. The differentiated retina occupies the layered and most central part of the tissue. Among the other retinal cell types, it contains PRCs and MG cells, which also express Rx2. Importantly, Notch signalling was not detected in RSCs. MG cells and PRCs did not show Notch signalling either (*n*=10 fish). This is in contrast to zebrafish, in which Notch signalling is active in MG cells (Wan and Goldman, 2017). The lack of overlap of Notch signalling with RSCs, MG cells or PRCs can be clearly appreciated in the *tp1::*d2GFP retina 3D reconstruction from a frontal (Fig. S1A,A′) and lateral (Fig. S1B,B′) view. Our data show that Notch signalling is confined to a subset of progenitors in the CMZ of the medaka retina.

### Notch-positive progenitors give rise to MG cells, ACs and BCs

Notch signalling has been previously shown to be involved in cell-fate choices in the embryonic vertebrate retina (Andreazzoli, 2009; Livesey and Cepko, 2001). During post-embryonic retinal growth, we hypothesize lineage specification to occur in the transit-amplifying zone of the CMZ, between RSCs and the more centrally located terminally differentiated retina. Factors involved in lineage specification during retinal development, such as *atoh7* and *neuroD*, have been shown to be expressed in that zone (Lust et al., 2016; Poggi et al., 2005; Raymond et al., 2006; Taylor et al., 2015). Consistently, the activation of Notch signalling in the transit-amplifying zone of the CMZ points towards a role for Notch signalling in cell-fate specification during post-embryonic growth. Thus, we next addressed the differentiation potential of the Notch-positive progenitor pool.

To assess this, we crossed the *tp1::*tagRFP reporter line to the *tp1::*d2GFP reporter. In the retina of these double-transgenic fish, we distinguished two cell populations. One population, located towards the periphery of the CMZ, was double positive reflecting actual Notch signalling activation (Fig. 1G-G″, insets). The second population, located more centrally in the differentiated retina, was only positive for tagRFP and corresponds to cells derived from a Notch-positive progenitor pool (Fig. 1G-G″). Interestingly, these cells were only located in the INL and were identified as BCs, MG cells and ACs based on morphology and position: BCs are located in the apical part of the INL; the nucleus of MG cells is elongated and located in the centre of the INL; ACs have a round nucleus and are located in the basal part of the INL (Fig. 1G″, asterisks and inset on the right). No tagRFP-positive nuclei were observed in the ONL or GCL. The tagRFP signal that can be observed in those layers corresponds to cellular projections of BCs and MG cells. In addition, we validated the identity of the descendants of Notch-positive progenitor cells by co-staining with cell type-specific markers (Fig. S2). Interestingly, we observed a longer activation of Notch signalling in cells that will become MG cells (Fig. S3). This points towards a conserved role for Notch signalling in specifying MG cell fate, as has been previously reported in zebrafish and mammals (Bernardos et al., 2005; Jadhav et al., 2006; Scheer et al., 2001; Vetter and Moore, 2001). However, in contrast to zebrafish and mammals, Notch signalling does not remain active in fully differentiated MGs in medaka. Taken together, these results show that the lineage of the Notch-positive progenitor pool comprises a subset of retinal cell types, belonging to the INL.

### Notch signalling activation and *atoh7* expression show mutually exclusive patterns in the progenitor area of the post-embryonic medaka retina

Notch-positive progenitors are committed to differentiate into BCs, MG cells and ACs. These progenitors comprise only a subset of progenitors in the CMZ and do not generate the complete spectrum of retinal cells types. Therefore, another pool of progenitors must give rise to RGCs, PRCs and HCs, complementing the Notch lineage.

The bHLH transcription factor Atoh7 is well known for its role during retinal development in vertebrates (Kay et al., 2001; Ohnuma et al., 2002). *atoh7* is expressed in the final divisions of retinal progenitors and is known to be necessary for their differentiation into RGCs. The lineage of Atoh7-positive retinal embryonic progenitors comprises RGCs, PRCs, ACs and HCs (Poggi et al., 2005). It has been recently shown that *atoh7* expression is not restricted to embryonic development; a subset of progenitors in the CMZ also expresses *atoh7* during post-embryonic growth. This pool of progenitors has the same potential as its embryonic counterpart (Lust et al., 2016).

**Fig. 2.**
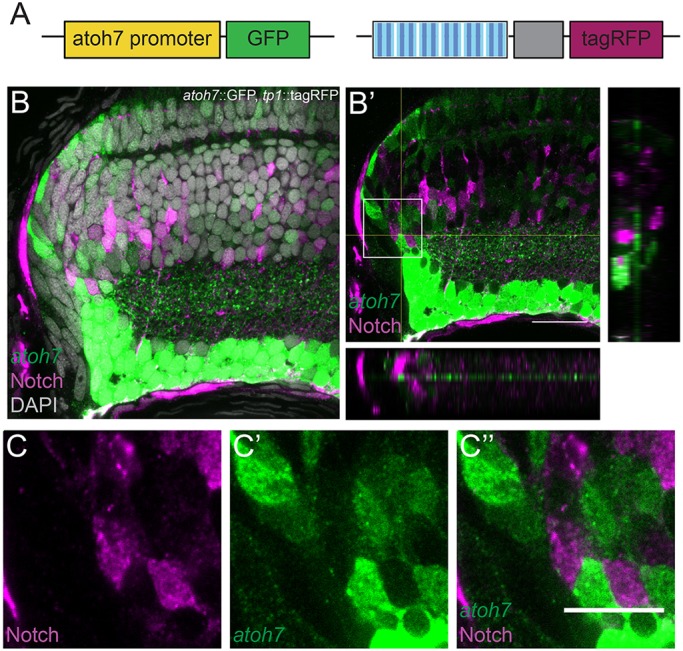
**Notch signalling activation and *atoh7* expression show a mutually exclusive pattern in the progenitor area of the post-embryonic medaka retina.** (A) The *atoh7* reporter line containing the *atoh7* promoter followed by a GFP was crossed to the *tp1::*tagRFP Notch reporter. (B,B′) Notch signalling activation (magenta) and *atoh7* expression (green) show mutually exclusive patterns in the progenitor area of the CMZ. DAPI is shown in grey in B. Orthogonal views (*xz*, *yz*) of the progenitor area in B′ are shown. (C-C″) Higher magnification of the progenitor area indicated with a square in B′. Notch signalling activation is shown in magenta (C), *atoh7* in green (C′) and the merge is shown in C″. *n*=8 fish. Scale bars: 20 μm (B′); 10 μm (C-C″).

In order to investigate how Atoh7-positive progenitors localize within the CMZ with respect to the Notch-positive progenitors, the *atoh7::*GFP reporter line (Lust et al., 2016) was crossed to the *tp1::*tagRFP reporter line (Fig. 2A). In double-transgenic fish, Notch- and Atoh7-positive progenitors showed a mutually exclusive pattern in the progenitor area of the CMZ: in that area of the retina, the progenitor cells were either Notch positive or Atoh7 positive (Fig. 2B-C″). The number of double Notch- and Atoh7-positive cells in the progenitor area of the CMZ was as low as 0.94±0.43% (s.d.) positive cells per fish (*n*=3 fish and 1119 cells). This mutually exclusive arrangement of Notch-positive and Atoh7-positive cells resembles the Notch-Delta lateral inhibition pattern previously described in numerous organisms and tissues based on RNA expression patterns (Chitnis et al., 1995; Kiernan et al., 2005; Parks et al., 1997). Interestingly, a third population of progenitors negative for both Notch and Atoh7 was also observed. These results indicate that Notch signalling and *atoh7* expression are tightly coordinated to form a mutually exclusive pattern. Moreover, the lineages derived from Notch- and Atoh7-postive progenitors show a striking complementarity. This led us to hypothesize that crosstalk between Notch signalling and *atoh7* expression might regulate cell fate and lineage restriction during post-embryonic growth.

### Toolbox for targeted activation of Notch signalling in Atoh7-positive progenitors

To address the role of the Notch-Atoh7 crosstalk in cell-fate specification in the retina, we decided to interfere with the tightly regulated balance in the progenitor area by activating Notch signalling in Atoh7-positive progenitors and analysing the effects on lineage specification.

**Fig. 3.**
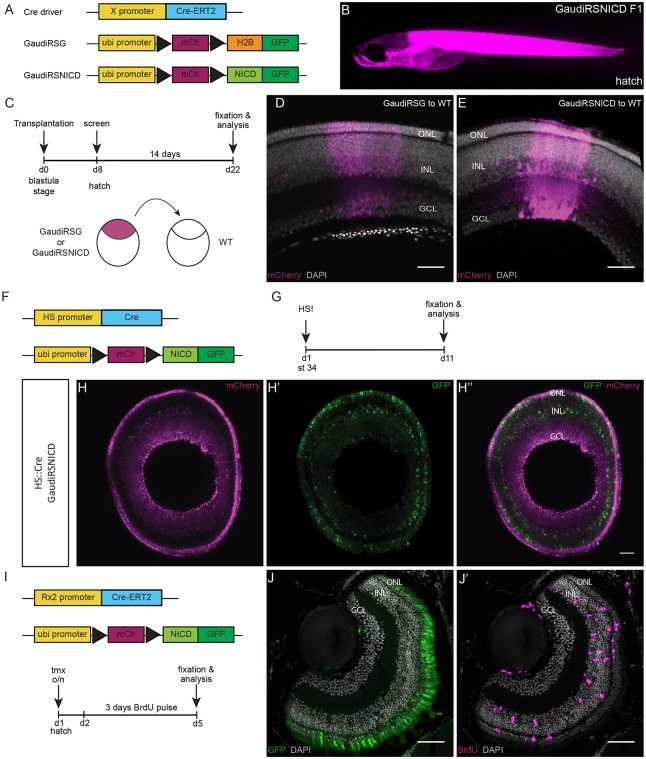
**The GaudíRSNICD line recombines ubiquitously and is functional.** (A) Specific promoters (X promoter) can be used to drive expression of an inducible Cre recombinase in specific cell populations to achieve targeted recombination of the GaudíRSG construct and the GaudíRSNICD. The GaudíRSG construct is the control construct: it switches from mCherry to H2B-GFP upon recombination. Black triangles represent LoxP sites. The GaudíRSNICD construct is the experimental construct: it switches from mCherry to Notch intracellular domain (NICD) fused to GFP upon recombination. (B) GaudíRSNICD fish after hatching showing ubiquitous mCherry expression (magenta). (C) Transplantations were performed at blastula stage (day 0, d0) from GaudíRSG or GaudíRSNICD embryos into wild-type (WT) embryos (see scheme below). The fish were screened for mCherry expression at hatch (d8) and grown for 14 days. After that, they were fixed and analysed (d22). (D,E) The transplanted cells from both lines (GaudíRSG in D and GaudíRSNICD in E) integrated in the host retina and differentiated into all cell types (Centanin et al., 2011). mCherry-positive cells are shown in magenta and nuclear labelling with DAPI in grey. The retinal cellular layers are also indicated. (F) GaudíRSNICD fish were crossed to a heatshock (HS)*::*Cre line. (G) The double-positive fish were heatshocked (HS!) at stage 34 (st 34) and fixed and analysed 10 days later. (H-H″) Recombined retinae show mCherry expression (magenta, H) and recombination in all three cellular layers (GFP-positive cells, green, H′). H″ shows the overlay of H and H′. The three retinal layers are indicated. (I) Recombination of the GaudíRSNICD construct was induced with an inducible Cre recombinase under the control of *rx2* promoter. After overnight tamoxifen induction, the fish were incubated for 3 days in BrdU and then fixed and analysed. (J) Recombination in *rx2*-expressing cells such as PRCs, which are located in the ONL, could be observed. (J′) Massive proliferation in the central retina was detected by immunostaining against BrdU (magenta) (*n*=7 fish). This result recapitulated the previously reported phenotype observed upon Notch activation in Rx2-positive cells (Lust et al., 2016). Scale bars: 40 µm.

To achieve this, we generated an *atoh7::*^ERT2^Cre line as a recombination driver (Fig. S3). This line was validated by colocalization of *atoh7* and *cre* transcripts in the progenitor area of the CMZ (Fig. S4A-A″). The *atoh7::*^ERT2^Cre line was also validated for recombination in the *atoh7* expression domain in the CMZ. To do this, we crossed this driver line with a control line carrying a construct that results in a colour switch (from mCherry to H2B-GFP) upon recombination (GaudíRSG line; Centanin et al., 2014) (Fig. 3A). Immediately after recombination, we observed GFP-positive cells in the *atoh7* expression domain in the CMZ, indicating specific recombination in Atoh7-expressing progenitors (Lust et al., 2016) (Fig. S4B-B″). In addition, we validated the specific activity of the *atoh7::*^ERT2^Cre in progenitor cells by lineage analysis. One month after recombination of GaudíRSG by induction of *atoh7::*^ERT2^Cre, we detected terminating clones in a ring around the embryonic retina (Fig. S4C-C″), identifying the initially targeted Atoh7-positive cells as progenitors with limited proliferative potential.

To activate Notch signalling in Atoh7-positive cells specifically, we combined the *atoh7::*^ERT2^Cre driver line with an experimental construct switching from ubiquitous mCherry to GFP fused to the Notch intracellular domain (GaudíRSNICD line; Fig. 3A). Whereas recombination of the control GaudíRSG construct only induces a colour switch, recombination of the GaudíRSNICD construct will initiate activation of the Notch signalling pathway in combination with a colour readout. Because the recombination occurs at the genetic level, the switch will result in permanent labelling, which allows the lineage to be followed in both conditions.

The GaudíRSNICD line was first validated for ubiquitous expression, recombination and functionality. Hatchling fish from this line showed ubiquitous mCherry expression (Fig. 3B). To further confirm the ubiquitous expression of the construct at cellular level in the retina, we performed transplantations at blastula stage from GaudíRSNICD into wild-type fish (Fig. 3C). As a control line for ubiquitous expression we used the previously characterized line GaudíRSG (Centanin et al., 2014). The transplanted fish were screened at hatching stage for red fluorescence in the eye and grown for 2 weeks. After that period of time, they were fixed and stained for mCherry (Fig. 3C). Both GaudíRSG and GaudíRSNICD generated arched continuous stripes (ArCoSs; Centanin et al., 2014) with mCherry signal in all three cellular layers (ONL, INL, GCL) and therefore in all retinal cell types (Fig. 3D,E, Movie 1). These results confirm that the line expresses mCherry ubiquitously and the construct is actively transcribed in all retinal cell types.

Secondly, the line was validated for recombination. Recombination was induced with Cre recombinase under the control of the heatshock (HS) promoter at embryonic stage 34 (Fig. 3F). The fish were subsequently fixed and analysed 10 days after recombination (Fig. 3G). The analysed retinae showed recombination in all three cellular layers of the retina (Fig. 3H-H″).

Finally, the line was validated for functionality. It has previously been demonstrated that Notch signalling activation in Rx2-positive cells triggers massive proliferation in the central differentiated retina, which is proliferatively inactive in wild-type controls (Lust et al., 2016). We used this effect to validate the GaudíRSNICD line. The GaudíRSNICD line was crossed to the *rx2::* ^ERT2^Cre line (Reinhardt et al., 2015) and the previously reported effects were reproduced (Fig. 3I-J′). Upon recombination, GFP was observed in Rx2-positive cells such as the PRCs located in the ONL (Fig. 3J). Massive proliferation was observed in the central retina (Fig. 3J′), reproducing the previously described phenotype. Together, these results show that the GaudíRSNICD line can be ubiquitously activated to full functionality.

### Targeted activation of Notch in Atoh7-positive progenitors shifts cell-fate ratios

**Fig. 4.**
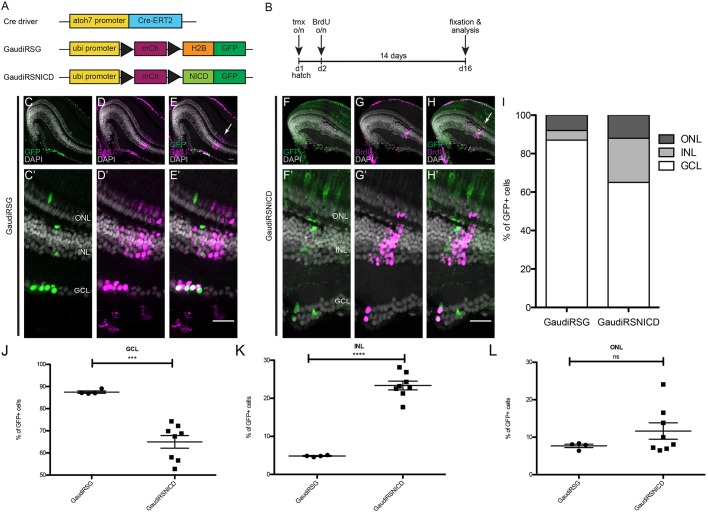
**Notch signalling activation in Atoh7-positive progenitors shifts cell-fate ratios during post-embryonic growth.** (A) The *atoh7* promoter was used to drive an inducible Cre recombinase exclusively in Atoh7-positive cells to achieve targeted recombination of the GaudíRSG construct (control construct: switch from red to green upon recombination) and the GaudíRSNICD construct (functional construct: switch from red to Notch intracellular domain with a GFP upon recombination). (B) Recombination was triggered with an overnight induction with tamoxifen at hatch. The fish were incubated in BrdU overnight to label the induction time point and grown for 14 days afterwards. At day 16 the fish were fixed and the lineage was analysed. (C-H′) GaudíRSG fish (C-E′) and GaudíRSNICD fish (F-H′) both show recombined cells (GFP positive, green) belonging to the Atoh7 lineage. These recombined cells are located at the BrdU (magenta) stripe or more peripherally. (I) Stacked column graph showing the percentage of cells in each layer derived from Atoh7-positive progenitor in control (GaudíRSG) and experimental (GaudíRSNICD) conditions. (J) Quantification of the percentage of GFP-positive (Atoh7-derived) cells shows a decrease in the GCL (***P=0.0003, *n*=1755 cells in 4 GaudíRSG retinae and 1428 cells in 8 GaudíRSNICD retinae). (K) Quantification of the percentage of GFP-positive (Atoh7-derived) cells shows an increase in the INL (****P<0.0001, *n*=100 cells in 4 GaudíRSG retinae and 522 cells in 8 GaudíRSNICD retinae). (L) Quantification of the percentage of GFP-positive (Atoh7-derived) cells remained constant in the ONL (*P*=0.2412, *n*=164 cells in 4 GaudíRSG retinae and 268 cells in 8 GaudíRSNICD retinae). ns, not significant. Error bars represent s.d. Scale bars: 20 μm.

After validation of the *atoh7::*^ERT2^Cre and the GaudíRSNICD lines, we addressed the impact of Notch-Atoh7 crosstalk on the specification of the resulting lineages. To that end, *atoh7::*^ERT2^Cre fish were crossed to GaudíRSG controls or GaudíRSNICD (Fig. 4A). Cre recombinase was activated at hatching stage by an overnight tamoxifen induction. The subsequent application of bromodeoxyuridine (BrdU) was used to leave a ‘timestamp’, highlighting the cells actively dividing at the time point of recombination. After a growth time of 2 weeks, the lineage was analysed by counting GFP-positive cells in each cellular layer located peripheral to the BrdU ‘timestamp’ (Fig. 4B).

Induction in the GaudíRSG control line confirmed the previously described lineage: Atoh7-positive cells give rise to RGCs, ACs, HCs and PRCs (Fig. 4C-E′, Fig. S5). Upon Notch activation, the same qualitative cell-type composition was observed in the GaudíRSNICD line (Fig. 4F-H′). However, the proportions of cell types generated in each condition were strikingly different (Fig. 4I-L). We observed a severe loss of RGCs in response to Notch activation in Atoh7-positive cells: the generation of cells from Atoh7-positive progenitors into the GCL was reduced from almost 90% to 65% (Fig. 4I,J). This was compensated for by a 5-fold gain in cell numbers in the INL (Fig. 4I,K). The generation of PRCs was not significantly altered by the activation of Notch signalling in Atoh7-positive progenitors (Fig. 4I,L). This change in cell-type ratios was not caused by an increase in cell death upon Notch activation (Fig. S6). In a scenario in which proliferation is altered, all cell types would be equally affected. Our results indicate that activation of Notch signalling shifts the proportions of cell types generated from the Atoh7-positive progenitors but it does not impact on their differentiation potential, suggesting a regulation of Atoh7 by Notch signalling. Thus, continuous activation of Notch signalling shifts cell-fate ratios within the Atoh7 lineage and leads to an increase in cells in the INL at the expense of RGCs.

### Inhibition of Notch signalling increases the number of Atoh7-positive progenitors

Because the activation of Notch signalling was able to shift the cell-fate ratio within the Atoh7 lineage, we investigated next whether Notch signalling was able to influence *atoh7* expression. This hypothesis was supported by previous results that had shown that the Notch targets Her factors negatively regulate bHLH transcription factors (Kageyama et al., 2007). Specifically in the retina, *atoh7* expression has been previously shown to be negatively regulated by Her factors during retinal development (Maurer et al., 2014; Schneider et al., 2001).

**Fig. 5.**
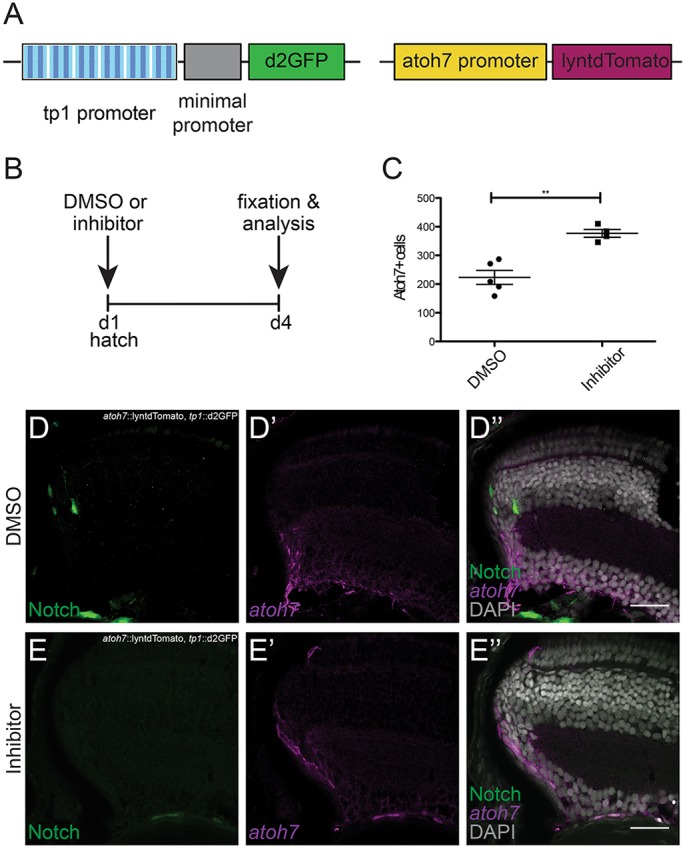
**Notch signalling inhibition increases the number of Atoh7-positive progenitors.** (A) Fish at hatching stage from a double reporter line carrying the *tp1::*d2GFP reporter and the *atoh7::lyntdTomato* reporter were used for this experiment. (B) The fish were treated with BrdU and DMSO (control) or Notch inhibitor (LY-411575) for 4 days. At day 4, the fish were fixed and analysed. (C) Quantification of Atoh7-positive cells (counted as lyntdTomato-positive cells) shows an increase upon Notch signalling inhibition (**P=0.001, *n*=1116 cells in 5 control fish and 1507 cells in 4 inhibited fish). (D-D″) Control fish show Notch signalling activation (GFP, green) and a restricted domain of Atoh7-positive cells (lyntdTomato, magenta). (E-E″) Notch-inhibited fish do not show Notch signalling activation and the *atoh7* expression domain is expanded. Error bars represent s.d. Scale bars: 20 μm.

To address whether Notch signalling regulates the expression of *atoh7* in the continuously growing medaka retina, we assessed the expression of *atoh7* upon Notch inhibition. To do so, we employed the Notch inhibitor LY-411575, which has been successfully used to block Notch signalling in zebrafish (Mizutari et al., 2013; Romero-Carvajal et al., 2015). We tested the drug on hatching-stage medaka and validated its specificity for the Notch signalling pathway in *tp1::*d2GFP reporter animals. Complete Notch signalling inhibition was achieved after 4 days of treatment (Fig. S7). Using BrdU and terminal deoxynucleotidyl transferase dUTP nick end labelling (TUNEL) staining, we excluded changes in proliferation and cell death in response to the treatment (Fig. S8). After validation of the inhibitor, we made use of double-reporter fish: *tp1::*d2GFP and *atoh7::*lyntdTomato (Fig. 5A). We incubated these double-reporter fish at hatchling stage with DMSO or Notch inhibitor for 4 days and fixed immediately after. These fish were analysed by immunostaining against the respective fluorescent proteins (Fig. 5B). Upon Notch inhibition, the number of Atoh7-positive cells in the progenitor area of the CMZ was almost doubled and the domain was more apically expanded (Fig. 5C-E″). This shows a repression of *atoh7* expression by Notch signalling in the post-embryonic medaka retina. Inhibition of Notch signalling de-represses *atoh7* expression and, consequently, more cells express *atoh7*, indicating the regulation of *atoh7* expression by Notch signalling.

### Notch inhibition blocks PRC generation and increases the number of cells in the INL

**Fig. 6.**
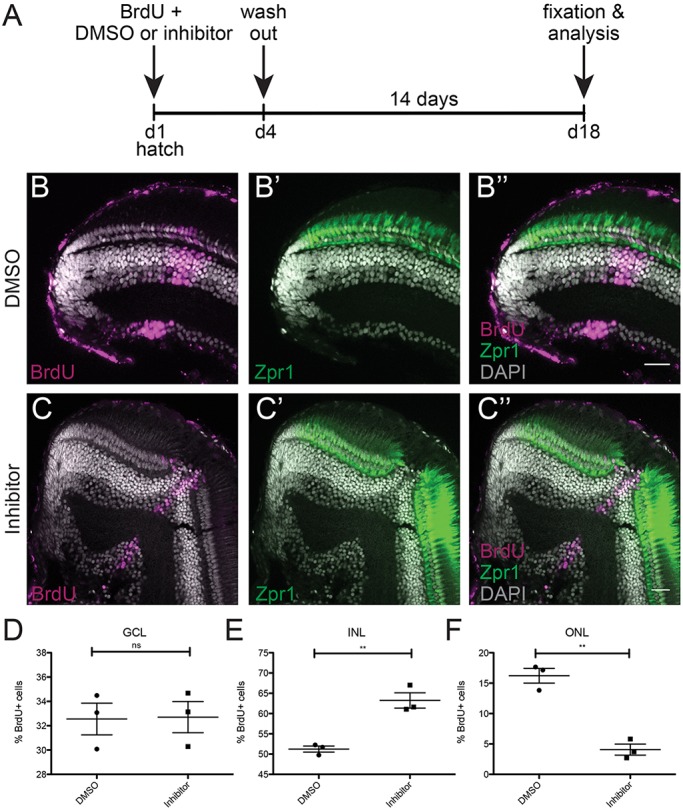
**Notch signalling inhibition shifts cell-fate ratios within the Atoh7 lineage.** (A) Wild-type fish were treated at hatch with BrdU and DMSO (control) or Notch inhibitor (LY-411575) for 4 days. At day 4, the BrdU and the DMSO or the inhibitor were washed out and the fish were grown for 14 days. At day 18, the fish were fixed and analysed. (B-B″) Control retinae display a normal morphology and the BrdU (magenta) stripe corresponding to the treatment time contains cells in the three cellular layers. The PRCs labelled with Zpr-1 (green) also display a normal morphology. (C-C″) Treated retinae display a disrupted morphology at the BrdU stripe, lacking the PRCs. (D-F) Quantification of BrdU-positive cells located in each layer in control conditions as well as in Notch-inhibited conditions reveals an increase in cells added to the INL and a decrease in photoreceptors located in the ONL (GCL: *P*=0.9372, *n*=710 cells in 3 control retinae and 1303 cells in 3 inhibited retinae; INL: **P=0.0042, *n*=1104 cells in 3 control retinae and 2443 cells in 3 inhibited retinae; ONL: **P=0.0013, *n*=334 cells in 3 control retinae and 169 cells in 3 inhibited retinae). Error bars represent s.d. Scale bars: 20 μm.

We next addressed the impact of Notch inhibition on the Atoh7 lineage. We treated wild-type fish at hatching stage for 4 days with either the LY-411575 inhibitor or DMSO as control and applied BrdU as timestamp. After 4 days, the treatment was stopped and the animals were placed back in normal fish water for 2 weeks. The fish were then fixed and the retinal cell-type composition analysed (Fig. 6A).

Control fish were unaffected and showed a morphologically normal retina with the three classical retinal layers both before and after the BrdU timestamp (Fig. 6B-B″). In contrast, the Notch-inhibited retinae showed a strong response to the absence of Notch signalling and the subsequent *atoh7* upregulation (Fig. 6C-C″). In the treated domain, marked by the BrdU timestamp, the retina lacked the PRCs in the ONL as shown by absence of the cone photoreceptor marker Zpr-1. Accordingly, Notch inhibition resulted in an increase in cell numbers in the INL. Quantification of BrdU-positive cells located in each layer in each condition showed a 4-fold decrease in cells of the ONL (PRCs absent) and a 123% increase in cells of the INL as a result of Notch inhibition (Fig. 6D-F). We cannot exclude the possibility that Notch inhibition, in addition to the Atoh7 lineage, might be impacting on other lineages or on the overall retinal polarity (Del Bene et al., 2008). Together with the results in response to the targeted activation of Notch, our data indicate that Notch signalling is necessary for achieving the proper cell-type proportions within the Atoh7 lineage. The fact that retinal growth and differentiation is properly re-instated after the extended and complete inhibition of Notch signalling indicates that the Atoh7-Notch pattern is re-established *de novo* and is crucial for the semi-crystalline architecture of the retina.

## DISCUSSION

Notch signalling is known to impact neural development greatly by influencing multiple aspects of this complex process (Louvi and Artavanis-Tsakonas, 2006). Several studies have previously addressed the role of Notch in the developing retina, as part of the nervous system (Del Bene et al., 2008; Perron and Harris, 2000). However, although expression of Notch pathway components has been reported in the CMZ of lifelong-growing organisms such as *Xenopus* and zebrafish (Dorsky et al., 1997; Raymond et al., 2006), its role in the continuous establishment of regularly patterned 3D neural columns of retinal cell types during continuous growth in the post-embryonic retina had not been addressed so far. Here, we employ two different Notch pathway sensors to show that Notch signalling is active in a regularly spaced subset of progenitors in the transit-amplifying zone of the CMZ in medaka. This population is fate-restricted to cells exclusively located in the INL: BCs, ACs and MG cells. In medaka, Notch signalling is active in a pattern that is mutually exclusive with the expression of the transcription factor Atoh7 in the transit-amplifying zone of the CMZ (Lust et al., 2016). Interestingly, this is an addition to the reported expression of Notch signalling components and *atoh7* in the CMZ of *Xenopus* (Perron et al., 1998). Atoh7-positive progenitors are fated to become RGCs, HCs, ACs and PRCs (Lust et al., 2016), showing a striking complementarity to the Notch lineage itself. Modulation and manipulation of Notch signalling by genetically targeted activation of the pathway in Atoh7-positive cells as well as by chemical interference demonstrate a crucial role for Notch signalling in generating the correct cell-type distribution and proportion within the Atoh7 lineage.

**Fig. 7.**
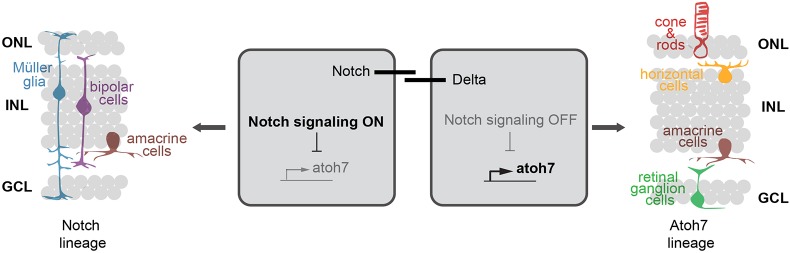
**Notch signalling regulates the expression of *atoh7* by lateral inhibition.** The cells that exhibit Notch signalling activation will repress *atoh7* expression and give rise to the cell types of the Notch lineage, namely MG cells, BCs and ACs (left). In the neighbouring cell, Notch signalling will be inactive and therefore *atoh7* can be expressed. Those cells will give rise to cell types of the Atoh7 lineage: PRCs, ACs, HCs and RGCs (right). Scheme adapted from Centanin et al. (2014).

The immediate impact of Notch signalling on *atoh7* expression in neighbouring cells in the post-embryonic retina and the resulting mutually exclusive pattern of expression/activity collectively argue for a lateral inhibition scenario. We propose that the Notch-Atoh7 pattern in the transit-amplifying zone of the CMZ shapes retinal architecture during continuous post-embryonic growth (Fig. 7): adjacent cells show an opposite status regarding Notch signalling activity. Active Notch signalling in one cell represses *atoh7* expression and fixes a range of cell types that Notch-positive progenitors give rise to (Notch lineage). Together with the descendants of the neighbouring Atoh7-positive cell (Atoh7 lineage), the full complement of retinal cell types can be established resulting in functional retinal columns in 3D. Strikingly, ACs are present in both lineages, consistent with the multiple subtypes of ACs that have been identified, suggesting that each lineage gives rise to different subtypes (Jusuf et al., 2011, 2012). Although the combination of both lineages covers all major retinal cell types, combining the two reporter lines carrying stable fluorophores, we observed cells in the central retina that were negative for both reporters. It is known that not only ACs but also several other retinal cell types can be further defined into subtypes (Jusuf et al., 2012; Kim et al., 2008; Schmidt et al., 2011; Suzuki et al., 2013; Wan and Goldman, 2017). Our data reveal retinal subtypes that are generated from neither Atoh7-positive nor Notch-positive progenitors. The presence of a third population of retinal progenitors in the CMZ, which was also negative for both Notch signalling and *atoh7* expression, supports the hypothesis of an additional mechanism to achieve the full spectrum of cellular diversity of the retina during post-embryonic growth, further extending the complexity of the lateral inhibition scenario.

Breaking the balance in the lateral inhibition scenario by activating or inhibiting Notch signalling has an impact on the Atoh7 lineage, indicating a crosstalk between Notch and Atoh7. Our results show that this crosstalk is accomplished by the repression of *atoh7* expression by Notch signalling. A direct repressive regulation of *atonal* genes by the Notch downstream factors (Her) has been previously shown in different model organisms and several tissues such as the inner ear, the lateral line and the intestine (Matsuda and Chitnis, 2010; Neves et al., 2013; VanDussen et al., 2012; Wilson et al., 2016). This regulation is a conserved mechanism to segregate lineages. In the inner ear of chicken, mouse and zebrafish, Notch signalling specifies the non-sensory lineage, which includes all support cells. In the neighbouring cells, Notch signalling is blocked by lateral inhibition and here *atoh1* expression specifies hair cell fate (Atkinson et al., 2015; Kiernan, 2013; Neves et al., 2013). Similarly, in the murine intestine, for example, Hes1 has been shown to promote the absorptive lineage by repressing *Atoh1* expression (Fre et al., 2005; Jensen et al., 2000). *Atoh1* is then expressed in the other intestinal lineage, the secretory lineage (Koch et al., 2013; VanDussen et al., 2012). Taken together, the Notch-*atonal* axis is a recurrently employed mechanism to segregate complementary lineages in complex 3D tissues.

In the embryonic retina, a regulation of proneural *atonal* genes by Notch signalling had been proposed in multiple model organisms based on modelling results and *in situ* analysis. Our results experimentally substantiate this proposed regulation of *atonal* genes by Notch signalling. We show that Notch signalling modulations immediately impact on the Atoh7 lineage by influencing *atoh7* expression. Modelling *Drosophila* eye development, it has been proposed that active Notch signalling inhibits R8 photoreceptor fate by inhibiting *atonal* genes within the same cell. In contrast, in the neighbouring cell, in which Notch signalling is laterally inhibited, *atonal* genes can be expressed allowing differentiation into R8 (Baker et al., 1996; Graham et al., 2010). In chicken, Notch signalling has been shown to inhibit RGC fate, arguing for an influence of Notch signalling on the Atoh7 lineage (Austin et al., 1995). Similarly, manipulations of Notch signalling in the developing mouse retina affect the cell types belonging to the Atoh7 lineage. With an inducible deletion of the Notch downstream effector RBPJκ, it was shown that autonomous RBPJκ inhibits RGC and photoreceptor fates (Riesenberg et al., 2009). Another study further supported regulation of *Atoh7* expression by Notch signalling in the developing mouse retina (Maurer et al., 2014). Conversely, the regulation of Notch by Atoh7 has not been addressed so far; however, the zebrafish mutant *lakritz*, which harbours a mutation in *atoh7*, displays peculiar retinal cell-type composition changes (Kay et al., 2001). The *lakritz* retina lacks RGCs but displays an increase in ACs, BCs and MG cells, which are the cell types we identified as the Notch lineage. It is tempting to speculate that Atoh7 has a repressing effect on Notch signalling, which is released in in *lakritz* mutants, leading to an increase in cell types of the Notch lineage. Our direct modulation of Notch signalling and its impact on *atoh7* in conjunction with those data demonstrate that the Notch-*atonal* axis is fundamental for the segregation of retinal lineages to ultimately achieve the correct cell-type composition and pseudo-crystalline architecture of the retina.

To address the link between growth and continuous patterning of the differentiating retina, it was crucial to address whether the pattern established by the Notch-*atonal* axis is perpetuated from the already differentiated central retina and thus impacts on the newly forming retinal columns or whether it is *de novo* established in a continuous fashion in the retinal progenitor cells. Our experiments completely blocking Notch signalling allowed the existing, highly patterned central retina to be disconnected from the newly forming tissue originating from the distal CMZ. Strikingly, even though cells are disturbed in their fate, impacting on retinal lamination in the domain experiencing the Notch signalling block, the retina continues to grow and re-instates proper lamination and differentiation *de novo*. This uncovers an effective self-organization capacity that establishes retinal patterning in the growth zone of the fish retina. It remains to be addressed whether this is deterministically initiated via an asymmetric division generating a Notch-positive and Notch-negative cell or whether, alternatively, Notch-positive or -negative cells are stochastically initiated upon exit from the niche to eventually propagate a mutually exclusive pattern with Atoh7 to the progenitor population by lateral inhibition. These scenarios show striking parallels to the proposed self-organization driven by Notch-Delta lateral inhibition interactions in the sensory organ in *Drosophila* (Corson et al., 2017). In these diverse contexts, the Notch-*atonal* axis acts a fundamental and highly evolutionarily conserved mechanism combining pattern establishment and cell-lineage specification to shift the temporal specification axis into the third dimension of cell types arranged in the retinal column.

## MATERIALS AND METHODS

### Animals and transgenic lines

Medaka (*Oryzias latipes*) used in this study were kept as closed stocks in accordance with Tierschutzgesetz 111, Abs. 1, Nr. 1 and with European Union animal welfare guidelines. Fish were maintained in a constant recirculating system at 28°C on a 14 h light/10 h dark cycle (Tierschutzgesetz 111, Abs. 1, Nr. 1, Haltungserlaubnis AZ35–9185.64 and AZ35–9185.64/BH KIT). The following stocks and transgenic lines were used: wild-type Cabs, GaudíRSG (Reinhardt et al., 2015), *GaudíRSNICD*, rx2*::*^LoxP^N3ICD (Lust et al., 2016), *atoh7::*iCre, *rx2::*iCre (Reinhardt et al., 2015), *HS::Cre* (Centanin et al., 2014), *tp1-MmHbb::*d2GFP (Lust et al., 2016), *tp1-MmHbb::*tagRFP, *atoh7::*GFP (Lust et al., 2016), *atoh7::*lyntdTomato. All transgenic lines were created by microinjection with Meganuclease (I-SceI) in medaka embryos at the one-cell stage, as previously described (Thermes et al., 2002), except for *tp1-MmHbb::*tagRFP, which was created by microinjection with Tol2.

### BrdU incorporation

For BrdU incorporation, embryos were incubated in 2.5 mM BrdU (Sigma-Aldrich) diluted in 1× embryo rearing medium [ERM; 17 mM sodium chloride, 0.4 mM potassium chloride, 0.27 mM calcium chloride dihydrate, 0.66 mM magnesium sulfate heptahydrate (pH 7)] for the amount of time indicated in the respective experiment.

### Induction of Cre/lox system

For ^ERT2^Cre induction, embryos were treated with a 5 µM tamoxifen solution (Sigma-Aldrich) in 1× ERM overnight. For HS*::*Cre induction, stage 34 embryos were moved to room temperature where the medium was removed completely from the plastic dish and replaced with 42°C ERM. Immediately after, the embryos were placed in a 37°C incubator for 2 h. Finally, they were returned to 28°C.

### Inhibitor treatment

LY-411575 (Sigma-Aldrich) was dissolved in DMSO to a 50 mM stock concentration. The stock solution was diluted in 2.5 mM BrdU to reach the final working concentration of 5 μM. Hatching-stage fish were treated with 5 μM LY-411575 in BrdU for 4 days at 28°C in the dark. The solution was exchanged after 2 days.

### Immunohistochemistry on cryosections

Fish were euthanized using 1×Tricaine (Sigma-Aldrich) and fixed overnight in 4% paraformaldehyde (PFA) in 1× PTW [1× PBS (pH 7.3), 0.1% Tween] at 4°C. After fixation, samples were washed with 1× PTW and cryoprotected in 30% sucrose in 1× PTW. To improve section quality, the samples were incubated in a 1:1 mixture of 30% sucrose and Tissue Freezing Medium (Leica) for at least 3 days. Serial sections (16-µm thick) were obtained on a Leica CM3050 S cryostat. Sections were rehydrated in 1× PTW for 30 min at room temperature. Blocking was performed for 1-2 h with 10% normal goat serum (NGS; Sigma-Aldrich) in 1× PTW at room temperature. Primary antibodies were applied at 1:500 in 1% NGS overnight at 4°C. Secondary antibodies were used at 1:750 in 1% NGS together with DAPI (Roth; 1:500 in 1× PTW of 5 mg/ml stock) and applied for 2 h at 37°C. Slides were mounted with 60% glycerol and kept at 4°C until imaging.

### BrdU immunohistochemistry on cryosections

BrdU antibody staining was performed with an antigen retrieval step. After all antibody and DAPI staining, except for BrdU, were complete, a 30 min fixation was performed with 4% PFA. Slides were incubated for 1 h at 37°C in 2 N HCl solution, and pH was recovered by washing with a 40% borax solution before incubation with the primary BrdU antibody.

### Immunohistochemistry on whole-mount retinae

Fish were euthanized using 20× Tricaine and fixed overnight in 4% PFA in 1× PTW at 4°C. After fixation, samples were washed with 1× PTW. Fish were bleached with 3% H_2_O_2_, 0.5% KOH in H_2_O for 2-3 h in the dark. Retinae were enucleated and permeabilized with acetone for 15 min at −20°C. Blocking was performed in 1% bovine serum albumin (Sigma-Aldrich), 1% DMSO (Roth/Merck), 4% sheep serum (Sigma-Aldrich) in 1× PTW for 2 h. Samples were incubated with primary antibody in blocking buffer overnight at 4°C. The secondary antibody was applied together with DAPI in blocking buffer overnight at 4°C. Primary antibodies were used at 1:200, secondary antibodies 1:250 and DAPI 1:500. The stained retinae were sectioned (40-μm thick) with the Vibratome Leica VT 1000S. Prior to sectioning, the retinae were embedded into 4% agarose (Sigma).

### Antibodies

The following primary antibodies were used: anti-EGFP (chicken; Life Technologies, A10262; 1:500), rabbit anti-Rx2 (Reinhardt et al., 2015; 1:500), anti-tagRFP (rabbit; Evrogen, AB233; 1:500), anti-Pax6 (rabbit; Hiss Diagnostics, PRB-278P; 1:200), PKCα (rabbit; Santa Cruz, sc-208; 1:200), anti-GS (mouse; Chemicon, MAB302; 1:500), anti-Sox2 (rabbit; Genetex, GTX101506; 1:500), anti-HuC/D (mouse; Thermo Fisher, A21271; 1:500), anti-recoverin (rabbit; Millipore, AB5585; 1:500), anti-Zpr-1 (mouse; Zebrafish International Resource Center; 1:500), anti-DsRed (rabbit; Clontech, 632496; 1:500), anti-BrdU (rat; AbD Serotec, BU1/75; 1:200). The following secondary antibodies were used: anti-mouse Cy5 (Jackson ImmunoResearch, 715-175-151), anti-chicken 488 (Jackson ImmunoResearch, 703-485-155), anti-rat DyLight549 (Jackson ImmunoResearch, 112-505-143), anti-rabbit DyLight549 (Jackson ImmunoResearch), anti-mouse Alexa546 (Life Technologies, A-11030) and anti-rat Alexa633 (Life Technologies, A21094). DAPI (Sigma-Aldrich, D9564) nuclear counterstaining was performed as described by Inoue and Wittbrodt (2011).

### Whole-mount double-fluorescence *in situ*

Whole-mount double-fluorescence *in situ* was performed with the TSA-Plus Cyanine 5 system from PerkinElmer as previously described (Reinhardt et al., 2015).

### TUNEL

TUNEL staining on cryosections was performed after all other antibody stainings were completed using the In Situ Cell Death Detection Kit TMR Red from Roche. Staining was performed according to the manufacturer's protocol with the following modification: washes were performed with 1× PTW instead of PBS.

### Immunohistochemistry and fluorescence *in situ* imaging

All immunohistochemistry and fluorescence *in situ* images were acquired by confocal microscopy using a Leica TCS SPE with either a 20× water objective or a 40× oil objective or a Leica TCS SP8 with 20× or 63× oil objective.

### Image processing and statistical analysis

Images were processed using Fiji image processing software. Statistical analyses and graphical representation of the data were performed using the Prism software package (GraphPad). Unpaired *t*-tests were performed to determine the statistical significances. *P*<0.05 was considered significant and *P*-values are given in the figure legends. Sample size (*n*) is mentioned in every figure legend. No statistical methods were used to predetermine sample sizes, but our sample sizes are similar to those generally used in the field. The experimental groups were allocated randomly, with no blinding during allocation.

## Supplementary Material

Supplementary information
