# Supplementary material for: Notch signalling patterns retinal composition by regulating atoh7 during post-embryonic growth
Source: Development. 2018 Nov 9;145(21):dev169698. doi: 10.1242/dev.169698 (PMC6240314; doi:10.1242/dev.169698)
Supplement: Supplementary information [file develop-145-169698-s1.pdf]

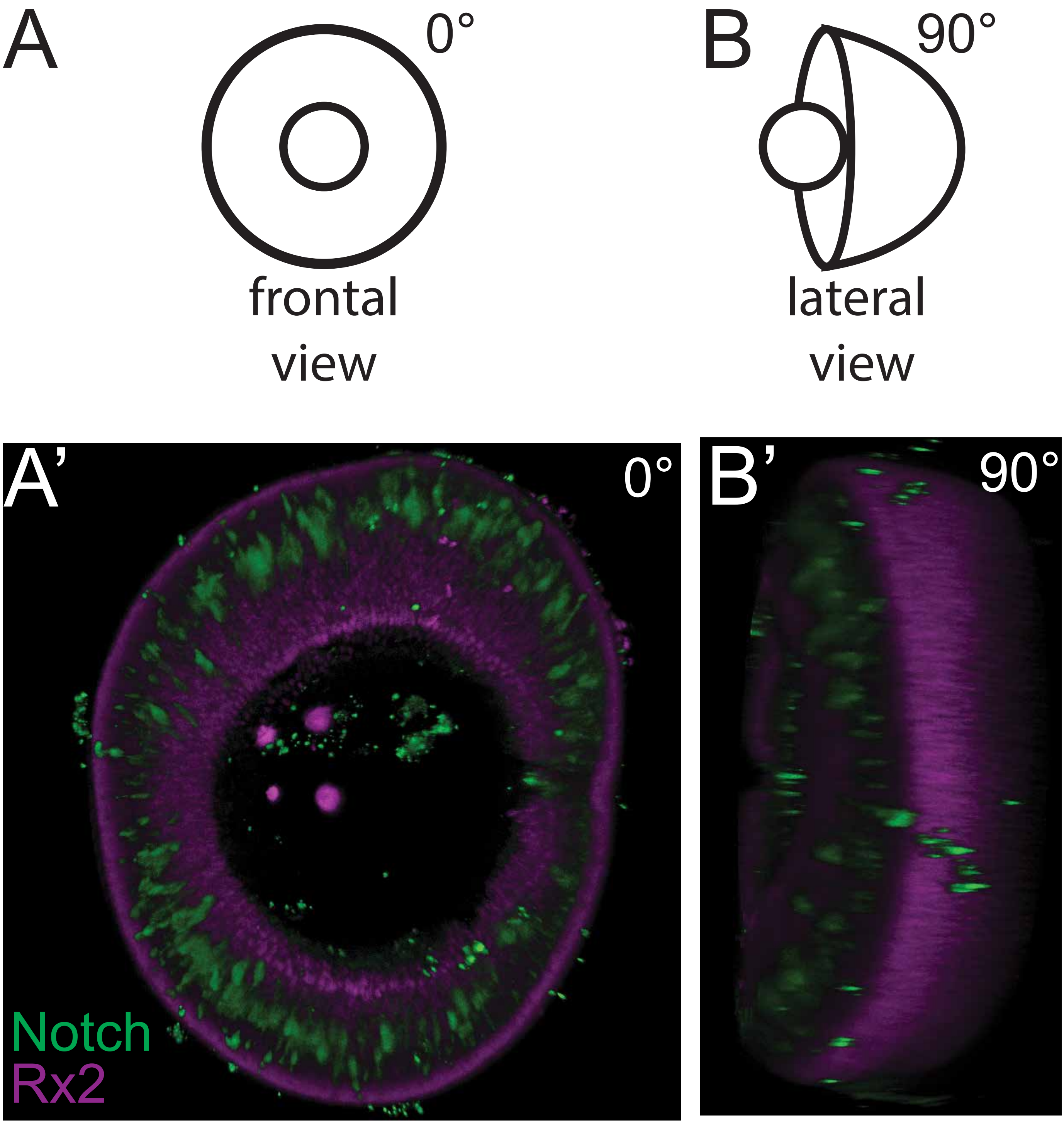

Figure S1. Notch signalling is not active in retinal stem cells in the postt embryonic retina in medaka. (A) Schematic representation of the retina from a frontal view (0°). (A') Frontal view of a retina 3D reconstruction. (B) Schematic representation of the retina from a lateral view (90°). (B') Lateral view of a retina 3D reconstruction. GFP is shown in green and Rx2 in magenta.

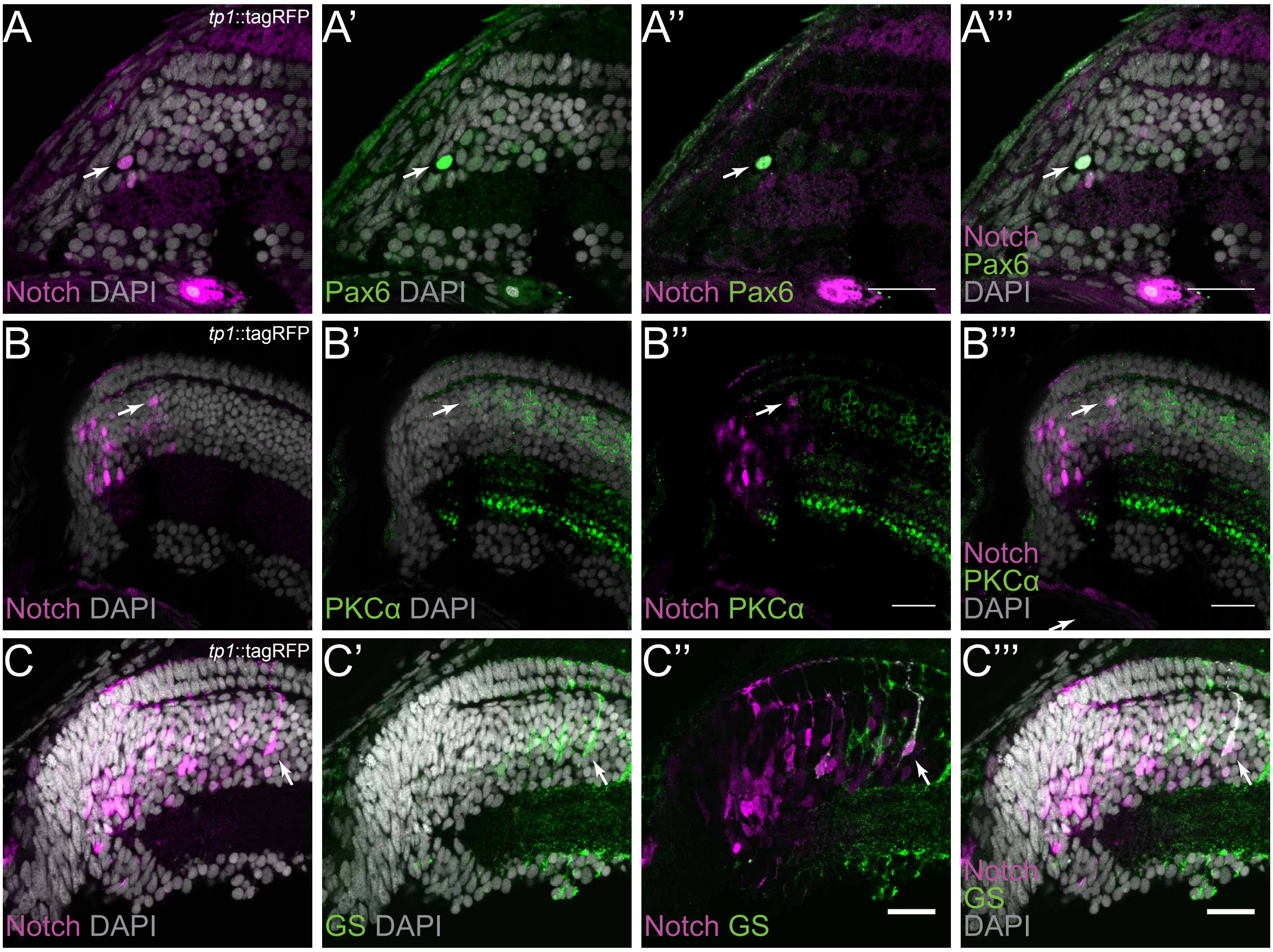

Figure S2. Notcht positive progenitors give rise to Müller glia cells, amacrine cells and bipolar cells. Endogenous tagRFP signal is shown in A and B (magenta). Immunostaining against tagRFP is shown in C (magenta). A', B' and C' show immunostaining against cell type specific markers (green). A'', B'' and C'' show the merge of tagRFP and the marker. In A''', B''' and C''' the merge together with DAPI nuclear labelling is shown. Scale bar is 20 µm. (A-A''') Notch positive progenitors give rise to amacrine cells, detected by immunostaining against Pax6. (B-B''') Notch positive progenitors give rise to bipolar cells, detected by immunostaining against PKCα. (C-C''') Notch positive progenitors give rise to Müller glia cells, detected by immunostaining against glutamine sythetase (GS).

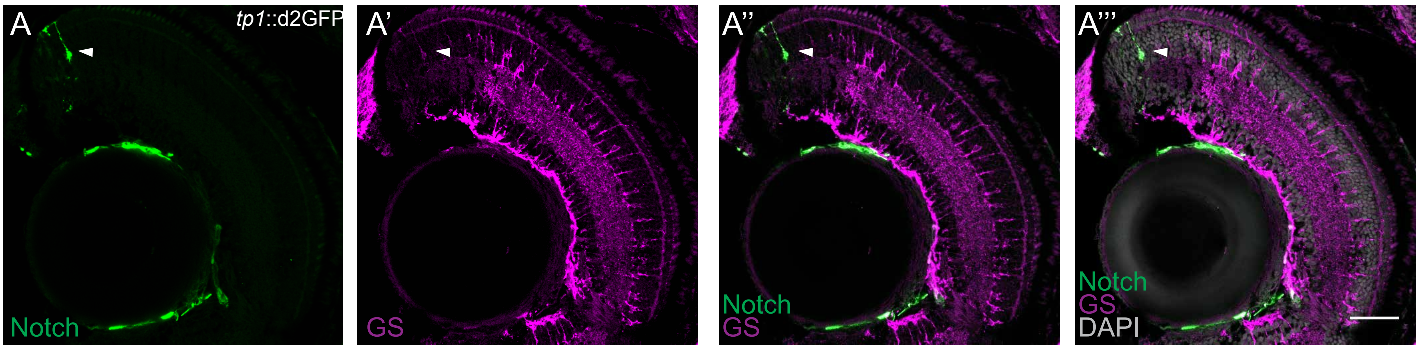

Figure S3. Notch signalling remains active in differentiating MG cells but it is switched off after complete differentiation. Cryosection of a *tp1*::d2GFP fish showing Notch signalling activity (A, green) in a differentiating MG cell, here identified by the upregulation of the MG marker glutamine synthetase (GS) (A', magenta). The co-localisation can be observed in the merge shown in A''. Nuclear labelling with DAPI is in grey (A'''). Scale bar is 40 µm.

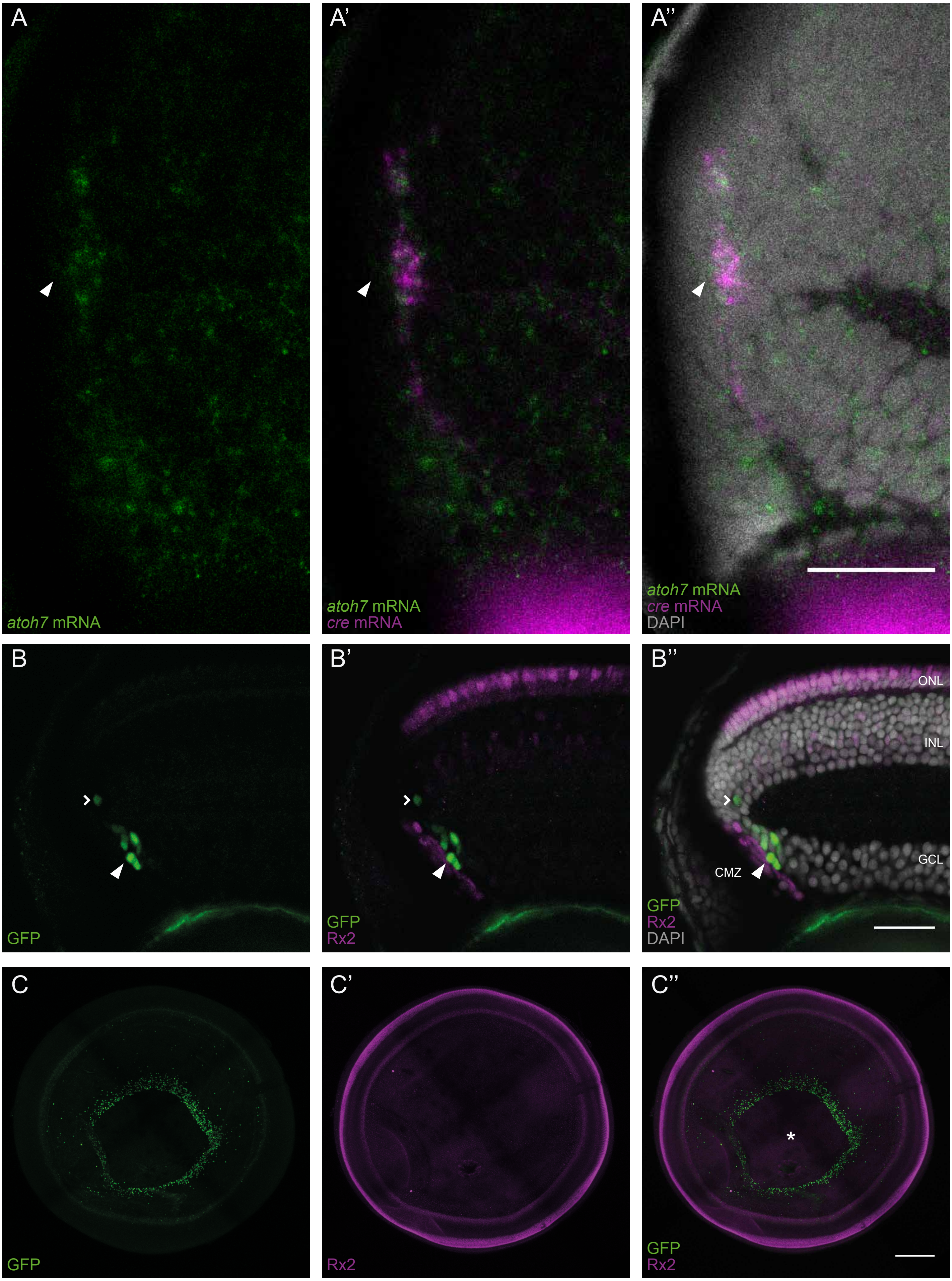
A
atoh7 mRNA
A'
atoh7 mRNA
cre mRNA
A''
atoh7 mRNA
cre mRNA
DAPI
B
GFP
B'
GFP
Rx2
B''
ONL
INL
GCL
CMZ
GFP
Rx2
DAPI
C
GFP
C'
Rx2
C''
*
GFP
Rx2

Figure S4. *atoh7*::^{ERT2}Cre induces recombination in progenitor cells of the retina. (At A'') Crossection of double fluorescent *in situ* hybridisation showing cot localisation of *atoh7* mRNA (A, green), *cre recombinase* mRNA (A', magenta) and the merge together with DAPI for nuclear labelling (A'', grey). The co-localisation is indicated with a white arrowhead. (B-B'') Cryosection of an *atoh7*::^{ERT2}Cre fish crossed to a GaudíRSG fish, induced at hatching and fixed 8 days later. Recombination (green) can be detected in cells located at the beginning of the GCL (arrowhead), directly adjacent to the *rx2* (magenta) expression domain. Few recombined cells are also detected close the beginning of the INL (open arrowhead). Nuclear labelling with DAPI is shown in grey. Scale bar is 25 µm. (C-C'') Maximum projection of a whole-mount immunohistochemistry of the retina of *atoh7*::^{ERT2}Cre fish crossed to GaudíRSG fish, induced at hatching and fixed 1 month later. Recombined cells (green) can be detected in a ring around the embryonic retina (asterisk). Scale bar is 200 µm.

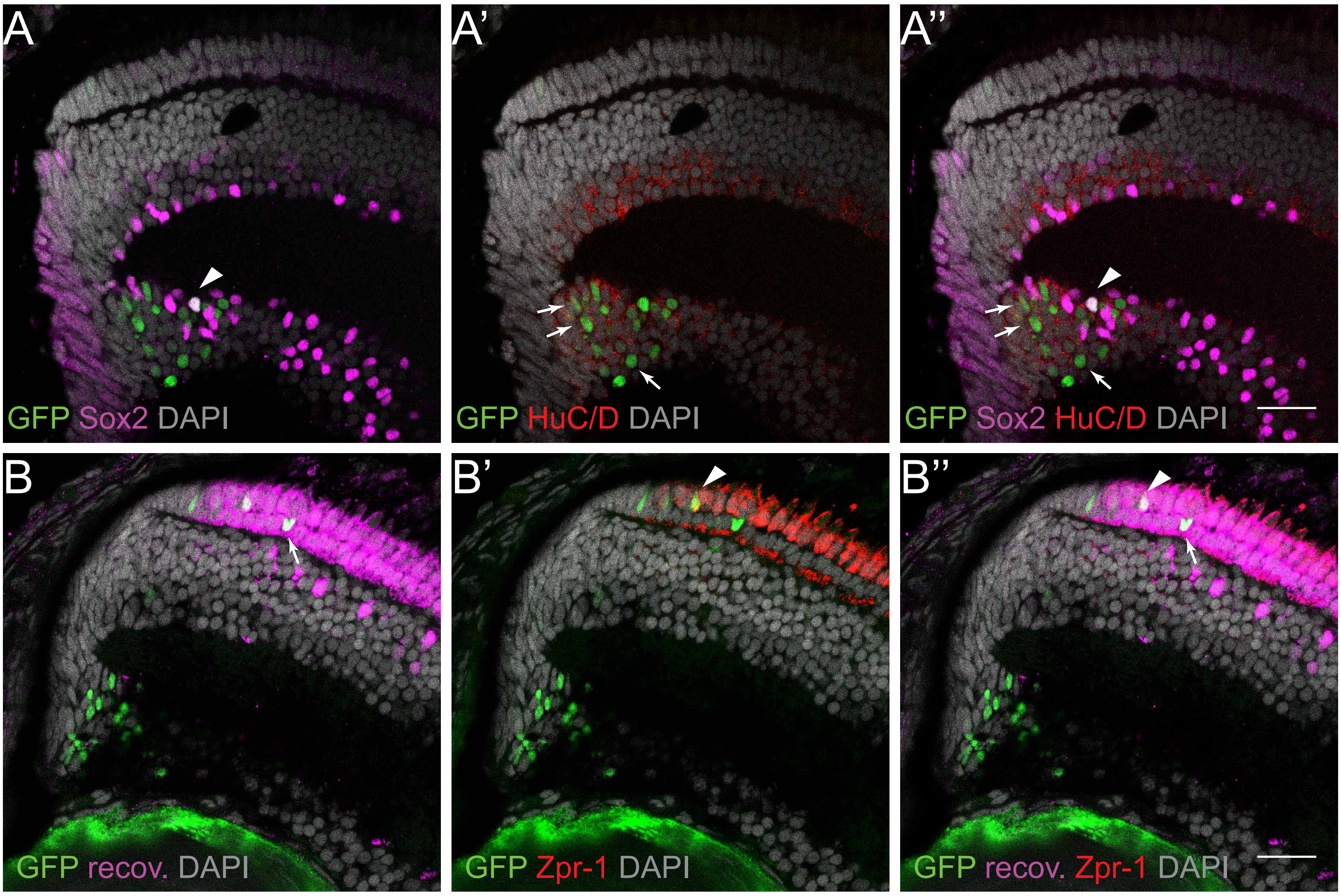

Figure S5. Atoh7 lineage. (Ai A'') Atoh7i positive progenitors can give rise to ACs and RGCs. ACs arising from Atoh7i positive progenitors were identified as GFPi positive cells that are also Sox2i positive and HuC/Di positive (indicated with an arrowhead on A). RGCs arising from Atoh7i positive progenitors were identified as GFPi positive cells that are HuC/Di positive and Sox2i negative (indicated with arrows on A'). A'' shows the merge. (Bi B'') Atoh7i positive progenitors can give rise to cone and rod PRCs. Cone PRCs arising from Atoh7i positive progenitors were identified as GFPi positive cells that are recoverini positive (indicated with arrows on A'). Rod PRCs arising from Atoh7i positive progenitors were identified as GFPi positive cells that are also Zpri 1 positive (indicated with an arrowhead on A). Nuclear labelling with DAPI is shown in grey. Scale bar is 20 µm.

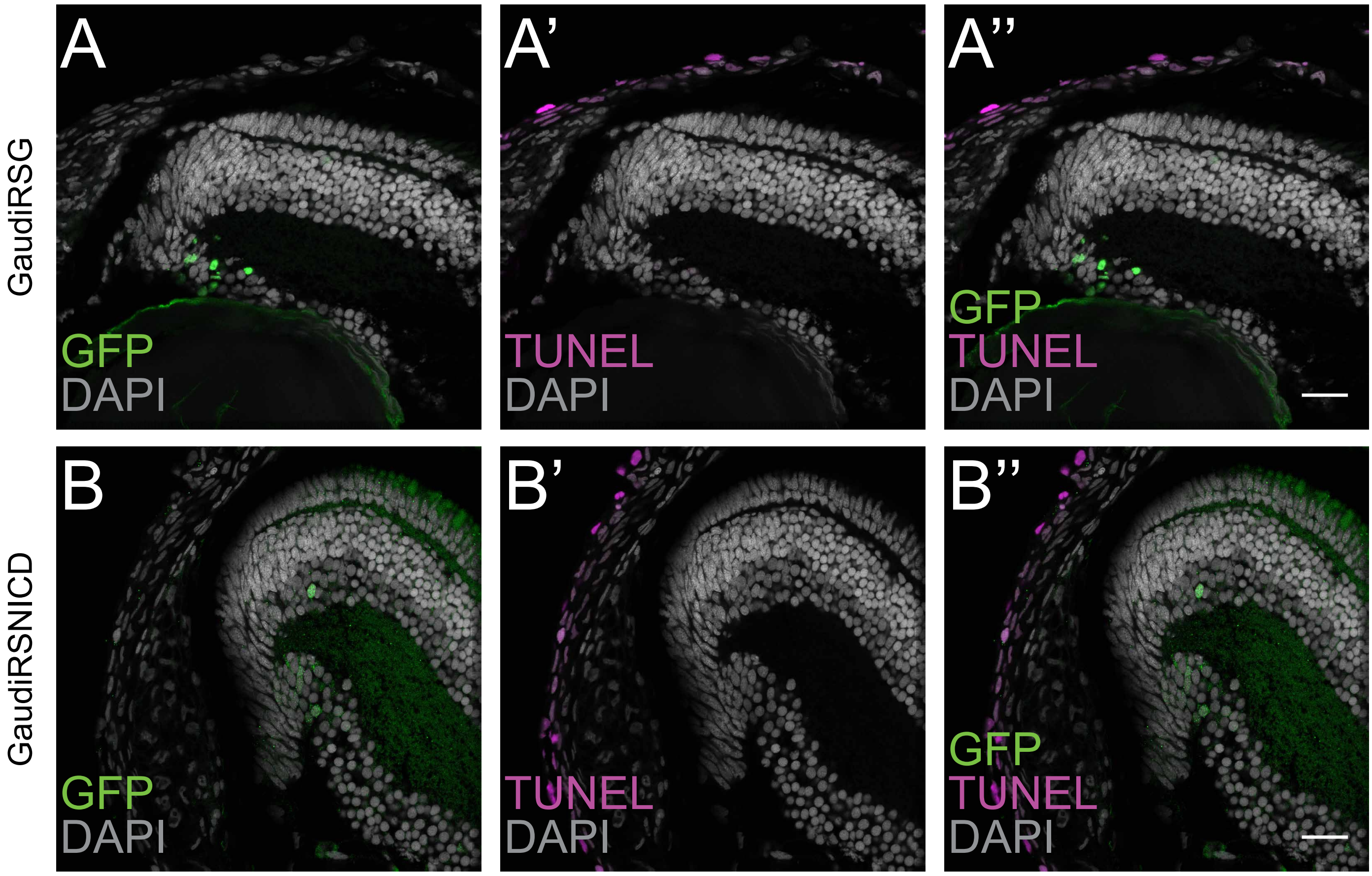

Figure S6. The GaudíRSG and GaudíRSNICD lines do not exhibit cell death in the retina upon recombination. Cryosection of an *atoh7*::^{ERT2}Cre fish crossed to a GaudíRSG fish (Aa A’’) or GaudíRSNICD fish (Ba B’’), induced at hatching and fixed 12 days later. Recombination can be detected by GFP expression (A, B, green, arrowhead). Cell death was detected by TUNEL staining (A’, B’, magenta). TUNEL-positive cells can be observed in the epithelium surrounding the eye (open arrowhead). The merge is shown in A’’ and B’’, respectively. Nuclear labelling with DAPI is shown in grey. Scale bar is 40 µm.

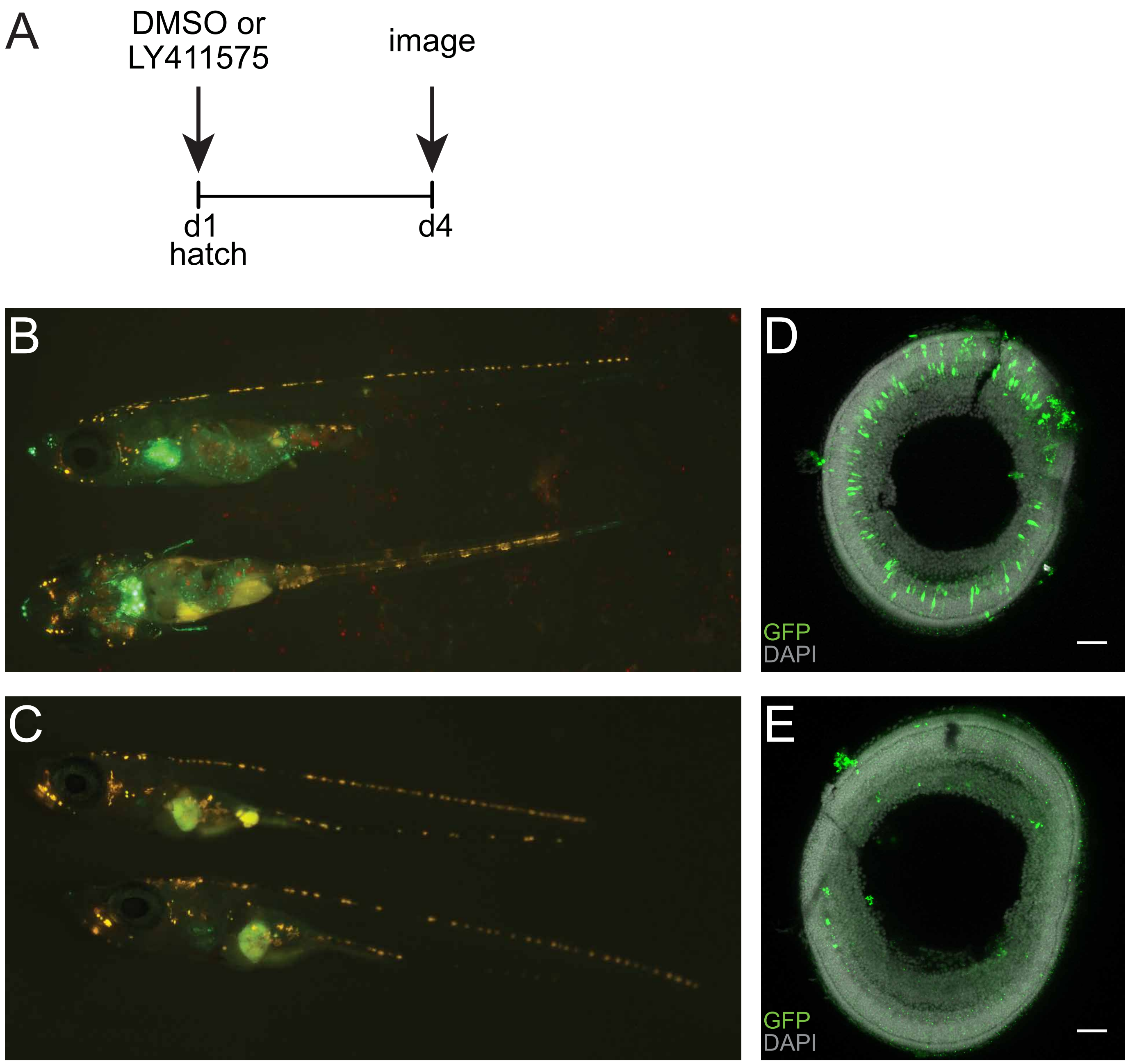

Figure S7. The inhibitor LY411575 inhibits efficiently Notch signalling. (A) *tp1::*d2GFP reporter hatch fish were incubated for 4 days in DMSO or 5 µM LY411575 and then either directly live-imaged or fixed and subsequently the retinae were dissected, immunostained for GFP and imaged. (B) The reporter fish show GFP fluorescence in Notch signalling active tissues when treated with DMSO. (C) After 4 days of inhibitor treatment, the fluorescence is clearly reduced and therefore Notch signalling is efficiently inhibited. (D) *tp1::*d2GFP retina treated with DMSO show Notch signalling activity in the progenitor area of the CMZ. (E) After 4 days of inhibitor treatment, the GFP signal is clearly reduced from the progenitor area of the CMZ demonstrating that Notch signalling activity in the retina is efficiently inhibited upon LY411575 treatment. D and E show maximum projections. Scale bar is 40 µm.

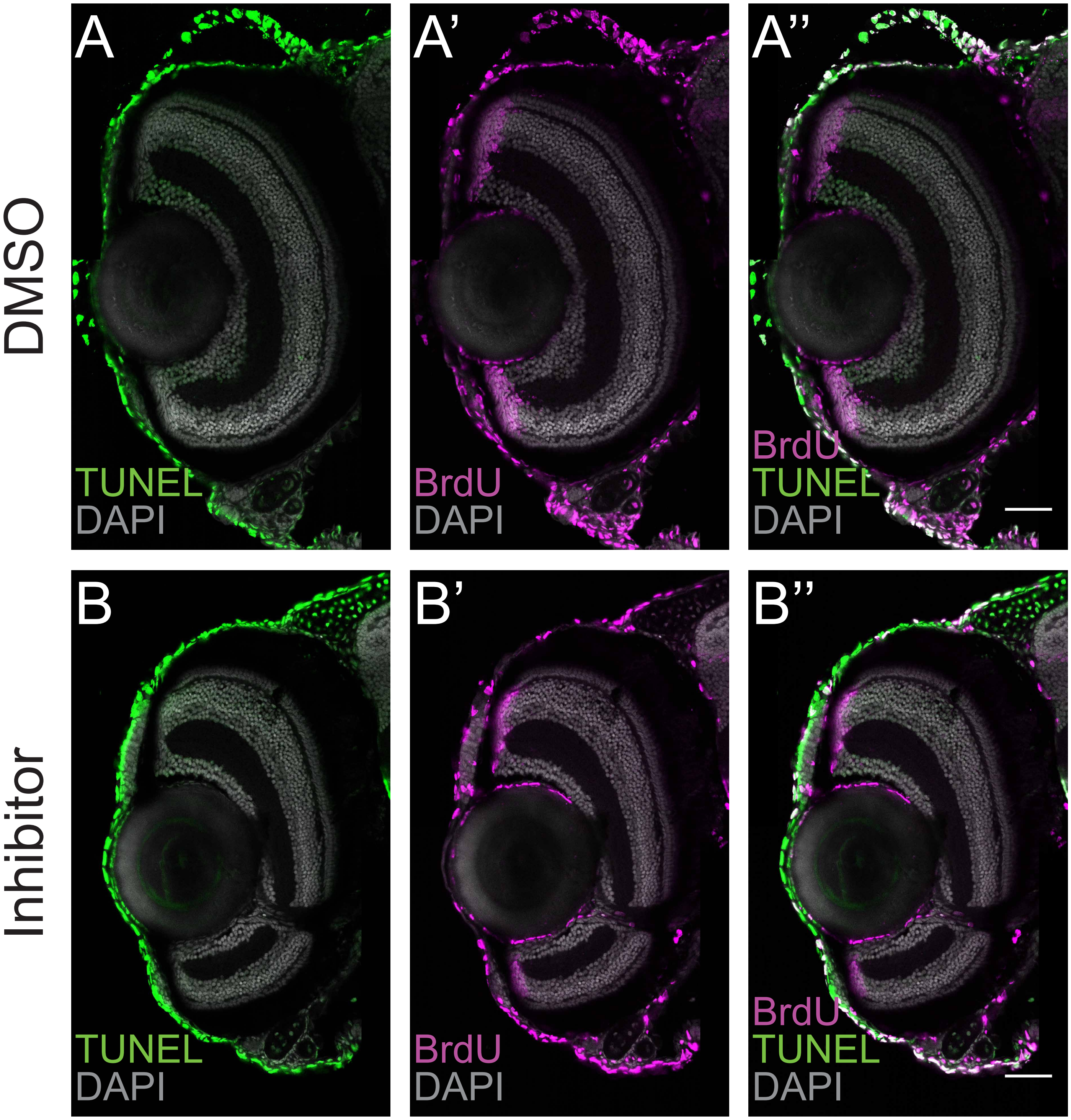

Figure S8. Treatment with the inhibitor LY411575 does not affect cell survival in the medaka retina. WT fish were treated with DMSO (Ad A’’, as control) or the inhibitor (Bd B’’) for 4 days together with BrdU. After that, the fish were fixed and immunostained for TUNEL (A,B, green) to detect cells death and BrdU (A’, B’ magenta) to analyse any changes in cell proliferation. The merge is shown in A’’ and B’’. Nuclear labelling with DAPI is shown in grey. Scale bar is 40 µm.

Development • Supplementary information

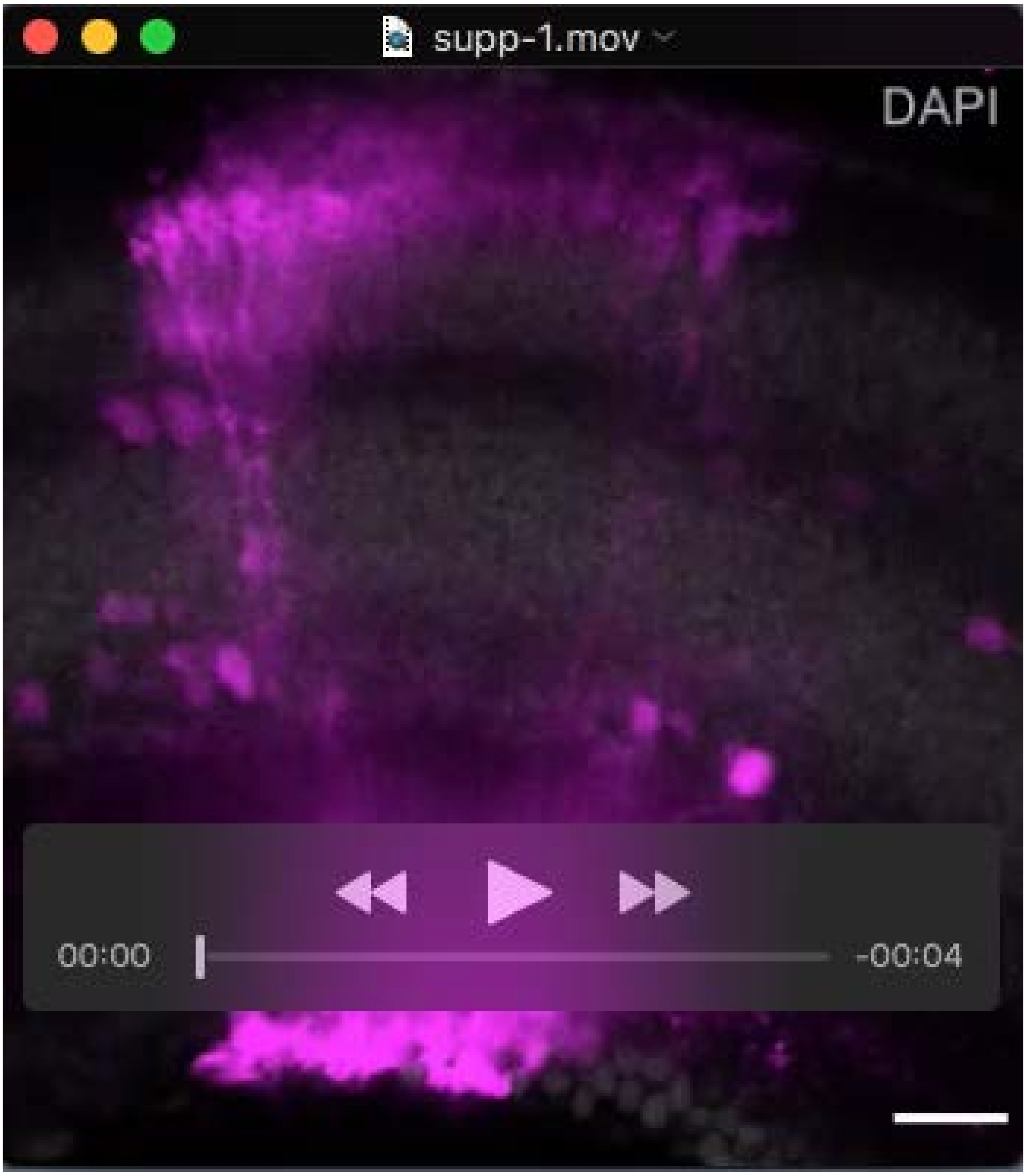

Movie 1. The transplanted cells from GaudíRSNICD (Fig. 3E) integrated in the host retina and differentiated into all cell types (Centanin et al., 2011), demonstrating the construct is ubiquitously expressed. The movie shows a z-stack. mCherry-positive cells are shown in magenta and nuclear labelling with DAPI in grey. Scale bar is 40 µm.
